# Communication from the cerebellum to the neocortex during sleep spindles

**DOI:** 10.1016/j.pneurobio.2020.101940

**Published:** 2021-04

**Authors:** W. Xu, F. De Carvalho, A.K. Clarke, A. Jackson

**Affiliations:** Institute of Neuroscience, Newcastle University, Newcastle NE2 4HH, UK

**Keywords:** Sleep, Cerebellum, Spindles

## Abstract

Surprisingly little is known about neural activity in the sleeping cerebellum. Using long-term wireless recording, we characterised dynamic cerebro-thalamo-cerebellar interactions during natural sleep in monkeys. Similar sleep cycles were evident in both M1 and cerebellum as cyclical fluctuations in firing rates as well as a reciprocal pattern of slow waves and sleep spindles. Directed connectivity from motor cortex to the cerebellum suggested a neocortical origin of slow waves. Surprisingly however, spindles were associated with a directional influence from the cerebellum to motor cortex, conducted via the thalamus. Furthermore, the relative phase of spindle-band oscillations in the neocortex and cerebellum varied systematically with their changing amplitudes. We used linear dynamical systems analysis to show that this behaviour could only be explained by a system of two coupled oscillators. These observations appear inconsistent with a single spindle generator within the thalamo-cortical system, and suggest instead a cerebellar contribution to neocortical sleep spindles. Since spindles are implicated in the off-line consolidation of procedural learning, we speculate that this may involve communication via cerebello-thalamo-neocortical pathways in sleep.

## Highlights

• We characterised dynamic cerebro-thalamo-cerebellar interactions during natural sleep in monkeys.
• Directed connectivity from motor cortex to the cerebellum suggested a neocortical origin of slow waves.
• Spindles were associated with a causal influence from the cerebellum to motor cortex, conducted via the thalamus.
• Dynamical systems analysis show that observed behaviour could only be explained by a system of two coupled oscillators.
• Results suggest a cerebellar contribution to neocortical sleep spindles.

## Graphical abstract

**Figure:**
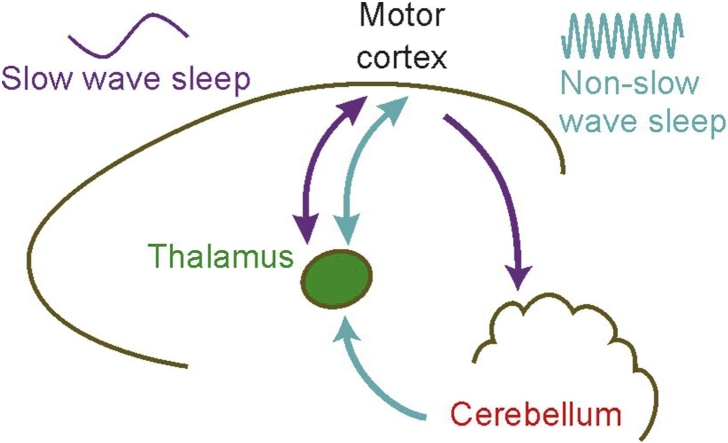

## 1 Introduction

The cerebellum plays a key role in motor learning (Bastian, 2006), while sleep is vital for consolidating and even enhancing new motor skills (Nishida and Walker, 2007; Diekelmann and Born, 2010; Fogel, Albouy et al. 2017). It is therefore surprising that the cerebellum remains an “uncharted land in sleep research” (Canto, Onuki et al. 2017). This oversight is due in part to the challenge of recording an electroencephalogram (EEG) signal from the intricate folds of the cerebellar cortex, with the absence of large surface potentials leading many animal sleep studies to use electrodes over the cerebellum as a reference, for example Latchoumane et al. (2017). In stark contrast to the wealth of literature implicating coupled sleep rhythms in the transfer of episodic memories from the hippocampus (Diekelmann and Born, 2010; Luthi, 2014; Staresina, Bergmann et al. 2015; Latchoumane, Ngo et al. 2017), little is known about oscillatory interactions between the neocortex and cerebellum during different sleep stages, or how these might contribute to off-line processing of procedural learning.

Cerebellar Purkinje cell firing rates are known to vary with sleep stage (Mano, 1970), and various features of the neocortical sleep EEG are correlated with fMRI signals in the cerebellum (Canto, Onuki et al. 2017). Of particular interest are sleep spindles, as these are associated both with cerebellar BOLD responses (Schabus, Dang-Vu et al. 2007; Fogel, Albouy et al. 2017) as well as off-line consolidation of procedural tasks such as motor sequence learning (Nishida and Walker, 2007; Diekelmann and Born, 2010; Fogel, Albouy et al. 2017). Spindles are waxing and waning 9−16 Hz oscillations thought to enhance neocortical plasticity (Niethard, Ngo et al. 2018). In a seminal series of experiments, Steriade and colleagues showed that thalamic spindles persist after decortication, whereas neocortical spindles are abolished by lesions of the thalamus and in particular the thalamic reticular nucleus (TRN) (Steriade, Deschenes et al. 1985). The hypothesis that sleep spindles arise in the TRN has been further supported by detailed descriptions of its intrinsic oscillatory properties (Steriade, Domich et al. 1987; von Krosigk et al., 1993). This would suggest that cerebellar activation associated with spindles reflects a downstream response, perhaps mediated via cortico-ponto-cerebellar pathways. However, since the deep cerebellar nuclei project directly to the thalamus, it remains possible that cerebellar output could modulate thalamo-cortical spindles (Schabus, Dang-Vu et al. 2007), thereby influencing neocortical learning processes during sleep. Due to the limited temporal resolution of the BOLD response, it is not possible to distinguish these possibilities with fMRI. Therefore we sought to characterise the presence and directionality of oscillatory cerebro-cerebellar interactions using intracranial signals recorded wirelessly during natural sleep in non-human primates.

## 2 Materials and methods

### 2.1 Subjects

Four female rhesus macaques (O: 11 y/o, 8.2 kg; U: 7 y/o, 7.7 kg; T: 10 y/o, 7.8 kg, Y: 6 y/o, 6.9 kg housed in pairs) were used for this study. Experimental objectives and procedures were approved by the local Animal Welfare Ethical Review Board and licensed by the UK Home Office in accordance with the Animals (Scientific Procedures) Act 1986 (2013 revision).

### 2.2 Electrode implantation

Surgeries were performed under sevoflurane anesthesia with postoperative analgesics and antibiotics. Animals were implanted with a head casing, and combinations of fixed, linear microelectrode arrays (16-channel LMAs, 12.5 μm platinum-iridium, 500 kΩ, MicroProbes for Life Sciences, USA) and individually-moveable, flexible microwires (50 μm Teflon-insulated tungsten, 200kΩ, Advent Research Materials, UK). We targeted the hand area of the primary motor cortex (M1), ventral-posterolateral (VPL) nucleus of the thalamus and contralateral lobule IV/V of the cerebellum as these are known to be anatomically connected (Kelly and Strick, 2003).

**Fig. 1:**
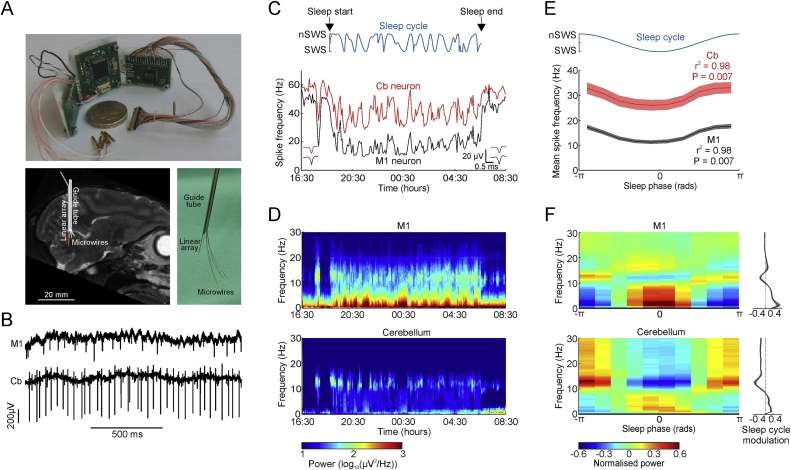
Long-term M1 and cerebellar recording during natural sleep. A. Wearable data-logging electronics and chronic cerebellar electrode implant for long-term recording during unrestrained behaviour. B. Example simultaneous recordings from primary motor cortex (M1, *top*) and cerebellum (Cb, *bottom*) during natural sleep. C. Firing rate of an example M1 (black) and cerebellar (red) neuron through a full night of sleep. Insets show averaged action potential waveforms from the first and last 1000 spikes in the recording. Blue trace plots depth of sleep calculated as negative cosine of sleep phase. D. Power spectrograms of M1 and cerebellar LFPs for the same session. E. Average spike firing rate against sleep phase for all M1 (black) and cerebellar (red) neurons in monkeys U and T combined. Shading indicates s.e.m. P and r^2^ values are from linear-to-circular correlation. Blue trace plots depth of sleep. F. Mean normalised power (proportional deviation from mean) as a function of sleep phase for all sessions with monkey U. Supplemental Fig. 3 shows data for all subjects. Side plots show sleep cycle modulation calculated from regression against cosine of sleep phase. Shading indicates s.e.m. Supplemental Fig. 3 shows corresponding plots for all animals.

For chronic M1 recordings, we implanted two LMAs (16 electrodes per shank, 0.5 mm spacing) and 12 microwires targeting the anterior bank of the central sulcus (visualised intraoperatively) at 16 mm lateral to the midline using techniques that we have described previously (Jackson and Fetz, 2007). For chronic cerebellar recordings, we implanted a single LMA targetting lobule IV/V at 7 mm lateral identified from individual MRI scans (Fig. 1A, Supp. Fig. 1). For spike recording in monkeys U and T, this was attached to the outside of a 16-gauge guide-needle within which 8 microwires were preloaded. Intraoperative recording from the LMA was used to verify penetration of the tentorium cerebelli, at which point the guide-needle was fixed and the microwires were lowered. Monkeys T and Y additionally received an LMA (16 electrodes, 0.6 mm spacing) targeting the VPL contralateral to cerebellar electrodes, verified intra-operatively from antidromic responses recorded on deep LMA contacts in the cerebellar nucleus.

### 2.3 Home-cage recording

During regular sessions under light sedation (generally at the start of each week), recording quality was assessed and microwires were moved manually to sample new neurons. We then mounted a custom battery-powered data-logger (Fig. 1A) for untethered recording in the animal’s home-cage (Xu, de Carvalho et al. 2019). The data-logger was based around two multichannel bio-amplifiers (RHD2132, INTAN Technologies, US) of which one captured spike data from selected microwires (typically 5 channels in M1 and 2 channels in the cerebellum; 0.1 Hz -7.5 kHz bandwidth, 20 kHz sampling rate) and the other recorded local field potentials (LFPs) from LMAs (16 channels in M1, 16 channels in the cerebellum, every other 8/16 channels in the thalamus; 0.1 Hz – 300 Hz bandwidth, 1 kHz sampling rate). Data was relayed to a 32GB microSD card by a low-power microcontroller (STM32 F407, STMicroelectronics, Switzerland). Recording sessions were initiated daily with a fresh battery and microSD card, yielding recordings that lasted around 20 h beginning with the animals awake and capturing a full night’s uninterrupted sleep.

### 2.4 Dataset

Our dataset comprised 145 sessions of LFP recordings from M1 and cerebellum (O: 48, U: 63, T: 29, Y: 5). M1 LFPs were taken from a deep, noise-free LMA channel, referenced to a local tungsten wire over the dura. Cerebellar cortex LFPs were taken from a noise-free channel near the middle of the LMA, re-referenced to the most superficial contact (below the tentorium).

In two animals (Monkeys T and Y) our recordings included thalamic LFPs taken from the deepest LMA contact (within the VLP thalamus) re-referenced to the most superficial contact (in the overlying white matter). We additionally analysed differential thalamic recordings taken from the deepest pair of adjacently recorded contacts within the VPL to exclude the possibility of volume conduction from distant sources.

In two animals (Monkeys U and T) we discriminated a total of 265 neurons in M1 (U: 235, T: 30) and 113 neurons in the cerebellum (U: 96, T: 17). Spike-spike analyses were based on 493 pairs of M1 and cerebellar neurons recorded simultaneously (U: 426, T: 67).

### 2.5 Data pre-processing

All off-line analyses were carried out with MATLAB (MathWorks USA). Filtering used 4-pole Butterworth filters applied in forward and reverse directions. LFP signals were down-sampled from 1000 Hz to 250 Hz after applying an anti-aliasing filter. Sleep start and end times were visually demarcated from spectrograms of M1 LFP (512 sample Hanning windows with 50 % overlap), and validated with simultaneous video recording in monkey O. Spikes were discriminated after high-pass filtering (300 Hz) using principal component analysis and clustering.

### 2.6 Sleep cycle architecture

We derived a continuous measure of sleep cycle phase from the envelope of low-frequency (<1 Hz) power modulations in M1 LFPs for the entire night (Xu, de Carvalho et al. 2019). The instantaneous low-frequency power through the night was low-pass filtered (cut-off frequency of 2.7 cycles/hour, roughly twice the duration of the natural sleep cycle of macaques) before a Hilbert transform was used to extract the sleep cycle phase.

We divided our continuous sleep phase into ten equal bins for subsequent cycle-aligned analyses of firing rates, LFP power spectra, coherence and directed coherence. To determine modulation through the sleep cycle, we calculated the slope of the regression between normalised power at each frequency and the cosine of sleep phase. Positive values indicated highest power during deep sleep while negative values indicated highest power during light sleep.

This continuous circular sleep phase was also used to identify periods of slow-wave sleep (SWS), based on the circular mean phase value in 30s-long windows. Sleep phases from – ^π^/_2_→0→^π^/_2_ corresponded to high <1 Hz power (SWS) while phases above ^π^/_2_ or below -^π^/_2_ corresponded to low <1 Hz power (non-SWS). We further subdivided non-SWS epochs into three categories: non-slow wave sleep containing spindles (nSWSs), non-slow wave sleep without spindles (nSWSns) and REM/arousal using the criteria described in the following two sections.

### 2.7 Criteria for identifying REM/arousal

In order to identify periods of REM and arousals we examined a previous dataset (33 sessions) gathered from monkey U in which M1 spike firing and electromyogram (EMG) from six contralateral arm muscles were recorded (Xu, de Carvalho et al. 2019). From this previous dataset we identified putative REM periods as any 30s-long window where spike firing rates exceeded mean waking level and where the average rectified EMG was below 2μV (Xu, de Carvalho et al. 2019). Additionally we identified 30s-long windows as ‘arousal’ if both spike firing rate and mean EMG were equal to or exceeded mean waking levels. Examination of the spectral properties of these REM and arousal windows revealed that both had significantly increased broadband M1 LFP power in the high-gamma band (50−125 Hz), which is known to reflect the firing rate of local neurons (Supp. Fig. 2A) (Nir, Fisch et al. 2007), and has previously been shown to be elevated during waking and REM in both humans (Cantero, Atienza et al. 2004) and rodents (Maloney, Cape et al. 1997). We derived a threshold on high-gamma power (normalised by its average through the night) based on receiver-operator curve (ROC) analysis (Supp. Fig. 2B, C). There are several ways of deriving the optimal threshold using the ROC curve (Habibzadeh et al., 2016). We employed 3 of them: 1) select the point on the curve closest to the top left corner, i.e. closest to the point of 100 % sensitivity and 100 % specificity; 2) select the point on the curve that intersects with the negative diagonal, i.e. where sensitivity equals specificity; 3) Youden’s index, i.e. the point that maximises the sum of sensitivity and specificity. The first two methods yielded the same point on the ROC curve (highlighted in red in Supp. Fig. 2C), the third method gives a point that correspond to a slightly lower threshold (highlighted in green in Supp. Fig. 2C). Therefore we took the mean of the corresponding thresholds of these two points (corresponding to 1.13 times the average high-gamma power through the night) and used this as our criterion for distinguishing REM/arousal epochs in our dataset. The proportion of REM/arousal periods derived using this method (Fig. 5A) is comparable to previously reported results for monkeys (Ishikawa, Sakai et al. 2017). Note that this method does not allow us to distinguish between REM and arousal states, but allowed us to exclude both from subsequent analysis of non-REM sleep.

### 2.8 Criteria for identifying sleep spindles and slow oscillations

Visual identification of spindles were carried out in accordance with criteria set out by the American Associate of Sleep Medicine (AASM) (Berry, Brooks et al. 2015). Automatic spindle detection was carried out using the method of Andrillon et al. (2011) (after first excluding REM/arousal epochs as described above), based on the magnitude of the analytic signal of band-pass filtered M1 LFP (9−16 Hz). A start-end threshold was set at one standard deviation above the mean magnitude (across the whole night), and a detection threshold was set at three standard deviations above the mean. Events were classified as spindles if the magnitude exceeded the start-end threshold for a period of more than 0.5 s and less than 2 s, and the peak magnitude exceeded the detection threshold. Events occurring within 1 s of each other were merged. Additionally, as per Andrillon et al. (2011), windows with high (>5 standard deviations) of 20−30 Hz power were removed, to ensure spectral specificity of spindle events. Throughout this paper, we use ‘spindle-band’ to refer to 9−16 Hz as per most human literature. However, for our dynamical modelling we use a 7−15 Hz band to best capture the empirically observed peaks in our data (see Section 2.11).

To identify up-states of the slow oscillation, we used a previously published method (Nir, Staba et al. 2011) whereby the LFP was first filtered at 0.5−4 Hz before identifying negative-going half waves with zero-crossings separated by between 0.25−1 s. As in that paper, we took the 20 % of events with the highest negative peaks, and compiled averages of neuronal firing rates and spindle occurrence aligned to these events.

Non-SWS epochs that were not classified as REM/arousal were divided into non-slow wave sleep without spindles (nSWSns) and non-slow wave sleep containing spindles (nSWSs), based on the presence of at least one identified sleep spindle event within a 30s-long window. While not identical to AASM criteria, these epochs are broadly comparable to stages N1 and N2 of sleep respectively. Supplemental Fig. 2D shows representative hypnograms for four animals based on these classifications.

We then analysed mean firing rates, LFP power spectra, coherence and directed coherence spectra separately for epochs classified as SWS, nSWSs, nSWSns and REM/arousal using a one-factor repeated measures ANOVA across sessions to test for modulation by sleep phase.

### 2.9 Coherence and directed coherence

Spike events were binned to produce time series with the same sampling resolution as LFPs (250 Hz). We then calculated LFP-LFP, spike-LFP and spike-spike coherence between the cerebellum and M1 according to:

(1) $${Coh}_{ij}(f)=\frac{{\left|P_{ij}(f)\right|}^{2}}{P_{ii}(f).P_{jj}(f)}$$

where $P_{ij}(f)$ is the cross-spectral density between channels i and j, and $P_{ii}(f)$ and $P_{jj}(f)$ are power spectra derived from non-overlapping 512 point windows (either through the entire sleep duration or within a single sleep phase). The 95th percentile significance threshold was calculated as:

(2) $$S=1-0.05^{1/(N-1)}$$

where N is the number of non-overlapping windows (Rosenberg, Amjad et al. 1989).

Directed coherence was derived using the method of spectral Granger causality. Bivariate autoregressive models was used to fit the LFP data (Schneider and Neumaier, 2001). To reduce computation time, we down-sampled the LFP by 3-fold to 83.3 Hz, and used a model order of 171 to maintain the same frequency resolution as the power and coherence spectra. Coefficients of the transfer matrix H(f) were obtained from inverting the spectral domain autoregressive coefficients (Kaminski, Ding et al. 2001). These were normalised using the method of Geweke (1982), as has been used previously with similar field potential data (Witham, Wang et al. 2010), yielding directed coherence:

(3) $$Dir{Coh}_{i⟵j}(f)=\frac{\left|H_{ij}\left(f\right)H_{ij}^{\text{*}}\left(f\right)C_{jj}\right|}{\left|H_{ij}\left(f\right)H_{ij}^{\text{*}}\left(f\right)C_{jj}+H_{ii}\left(f\right)H_{ii}^{\text{*}}\left(f\right)C_{ii}\right|}$$

where $C_{ii}$ and $C_{jj}$ are the covariances of the noise innovations of each signal in the autoregressive model. The directionality of interactions was assessed by comparing $Dir{Coh}_{i⟵j}$ versus $Dir{Coh}_{j⟵i}$ using a paired *t*-test across multiple sessions.

Additionally, we calculated coherence and directed coherence using 1 s-long windows moving in 0.1 s steps centred on the peak spindle amplitude. Significant modulation during the spindle was tested against high and low thresholds calculated as the mean ± 3 standard deviations obtained from two baseline ranges: between 2−3 s before the spindle event and between 2−3 s afterwards.

### 2.10 Spike firing parameters

For each neuron we measured the peak-to-peak spike width (Vigneswaran, Kraskov et al. 2011), mean firing frequency, and irregularity index of spike firing calculated according to Davies et al. (2006):

(4) $$Irregularity=\frac{1}{N-1}\sum_{i=2}^{N}\left|\text{l}\text{n}{(I}_{i}/I_{i-1})\right|$$

where and $I_{i}$ and $I_{i-1}$ denote consecutive interspike intervals. || denotes absolute value, and N is the total number of interspike intervals in the session.

### 2.11 Dynamical systems analysis

To examine systematic fluctuations in the relative phase of M1 and cerebellar oscillations, LFPs were band-pass filtered (7−15 Hz, capturing the peaks we observed in directed coherence spectra) prior to performing a Hilbert transform to extract the instantaneous phase-difference. These were binned into ten bins ranging from –π to +π (relative to the circular mean of the phase-difference throughout the night). We then calculated the average Hilbert amplitude of M1 and cerebellar oscillations, as well as the average of the amplitude time-derivative, separately for each phase-difference bin.

To explain these relationships, we sought to fit the observed LFPs using a variety of simple linear dynamical systems. Note that the Hilbert-transformed LFP provides both real and imaginary components, such that a single oscillator can be represented by a single complex state variable evolving according to:

(5) $$x\left(t+\Delta t\right)=A.x\left(t\right)+\varepsilon$$

where the complex coefficient, A, captures the dynamics (frequency and damping) of the single oscillator, and the complex noise innovation, ε, represents external inputs.

Two coupled oscillators can thus be represented by the following state equation:

(6) $$\left(\begin{array}{c} x_{M1}\left(t+\Delta t\right)\\ x_{Cb}\left(t+\Delta t\right) \end{array}\right)=\left(\begin{array}{cc} A_{M1} & A_{Cb\to M1}\\ A_{M1\to Cb} & A_{Cb} \end{array}\right).\left(\begin{array}{c} x_{M1}\left(t\right)\\ x_{Cb}\left(t\right) \end{array}\right)+\varepsilon (t)$$

where the dynamics matrix now contains complex coefficients reflecting the intrinsic oscillators ($A_{M1}$, $A_{Cb}$) as well as the strength and phase-delay of coupling in each direction ($A_{M1\to Cb}$, $A_{Cb\to M1}$). We determined these eight (four real and four imaginary) free parameters by regressing the real and imaginary components of the Hilbert-transformed LFPs against the same signals shifted by one sample point. Note that this analysis is conceptually similar to the auto-regressive models used for directed coherence, but we have replaced a high-order model in the time domain with a single-order model for a single frequency component by exploiting the analytic signals obtained from the Hilbert transform.

The complex noise innovation vector, ε(t), represents the real and imaginary components of each analytic signal that cannot be explained by the past-history of the system. We modelled these as white noise inputs with a covariance equal to the covariance of the (complex) residuals. This added a further four parameters to the model (two real variances and one complex covariance capturing common inputs).

We then simulated the behaviour of this system under white noise inputs. Simulated LFPs were taken from the real components of the two state variables, $\;x_{M1}$ and $x_{Cb}$, which were filtered and processed in the same way as the actual data.

To demonstrate that all components of our model were necessary to explain the data, we also compared five reduced models. Model 1 (common oscillatory input) assumed the system could be described by a single oscillator (Eq. (5)) with dynamics fit to the first (complex) principal component of the two analytic LFP signals. The remaining models were variants of Eq. (6), but with one or more coefficients set to zero and excluded from the regression:

Model 2 (uncoupled oscillators): $A_{M1\to Cb}=0$, $A_{Cb\to M1}=0$

Model 3 (M1 to Cb coupling): $A_{Cb\to M1}=0$

Model 4 (Cb to M1 coupling): $A_{M1\to Cb}=0$

In all cases, the covariance structure of the system inputs was determined from the covariance of the residuals after fitting.

We performed two statistical tests to establish that the full model performed better than any of the reduced models. First we used ten-fold cross-validation within each session to quantify goodness of fit using the mean-squared residual (MSR). We used a paired *t*-test (over sessions) to compare the MSR for each reduced model to the full model. Note that cross-validation is essential since the models have different numbers of free parameters, but this approach avoids concerns of overfitting since MSR is assessed on data not used to build the model, and its variability is assessed across independent sessions. Second, we quantified how well the simulated data reproduced the shape of relationships between phase-difference, amplitude and amplitude-derivatives, using a Pearson’s correlation coefficient, *R*. Again we used paired t-tests (over sessions) to compare *R-*values for each of the reduced models against the full model.

## 3 Results

### 3.1 M1 and cerebellum exhibit similar sleep cycles

To investigate cerebro-thalamo-cerebellar interactions in natural sleep, we used a custom neural data-logger for untethered recording from anatomically connected regions of the hand area of the primary motor cortex (M1), thalamus and cerebellum (Kelly and Strick, 2003). In four monkeys, we collected a total of 145 sessions comprising approximately 20 -h periods of unrestrained home-cage behaviour and natural sleep (Fig. 1A,B). Local field potentials (LFPs) were obtained from linear arrays implanted through cerebellar lobules IV/V (referenced relative to the most superficial channel below the tetorium), in contralateral primary motor cortex (M1, referenced relative to the cortical surface) and, in two animals, the ventral-posterolateral (VPL) nucleus of the thalamus (differential recording). In two animals we additionally recorded simultaneous spiking activity from M1 and cerebellum using moveable microwires.

Firing rates of both M1 and cerebellar neurons were generally lower in sleep than during the day, but activity in both areas fluctuated with a period of approximately 1 hour (Fig. 1C), matching the known sleep cycle in monkeys (Xu, de Carvalho et al. 2019). Cyclical modulations were also present in the M1 LFP power spectrogram (Fig. 1D, *top*) for 0−4 Hz (encompassing slow wave and delta bands) and 9−16 Hz (encompassing spindle) frequencies. By comparison, modulations of LFP power in the cerebellum during sleep were less apparent upon visual inspection of the spectrogram (Fig. 1D; *bottom*). Therefore, we extracted the phase of the sleep cycle based on the envelope of low-frequency neocortical LFP power, using a method which we have shown previously to provide a simple and robust quantification of sleep cycles (Xu, de Carvalho et al. 2019). Average firing rates in both M1 and cerebellum were significantly modulated through the sleep cycle (Fig. 1E; circular-linear correlation, r^2^ = 0.98, P = 0.007 both for M1 and cerebellar neurons) with the lowest activity at the phase of maximal slow-wave activity (slow-wave sleep, SWS) and the highest at the phase of minimal slow-wave activity (non-SWS). Sleep cycle-averaged LFP power spectra for M1 revealed a characteristic reciprocal pattern (Fig. 1F, *top*), with highest slow-wave/delta power during SWS, and highest spindle power during non-SWS. Despite the lower amplitude of cerebellar LFPs, sleep cycle-averaged power spectra revealed clearly the same reciprocity between these frequency bands (Fig. 1F, *bottom*, and Supp. Fig. 3). Thus, not only is the cerebellum active during sleep, but this activity also bears a striking resemblance to the sleep architecture of neocortex.

### 3.2 M1 and cerebellar LFPs exhibit phase-coupling at slow-wave and spindle-frequencies

**Fig. 2:**
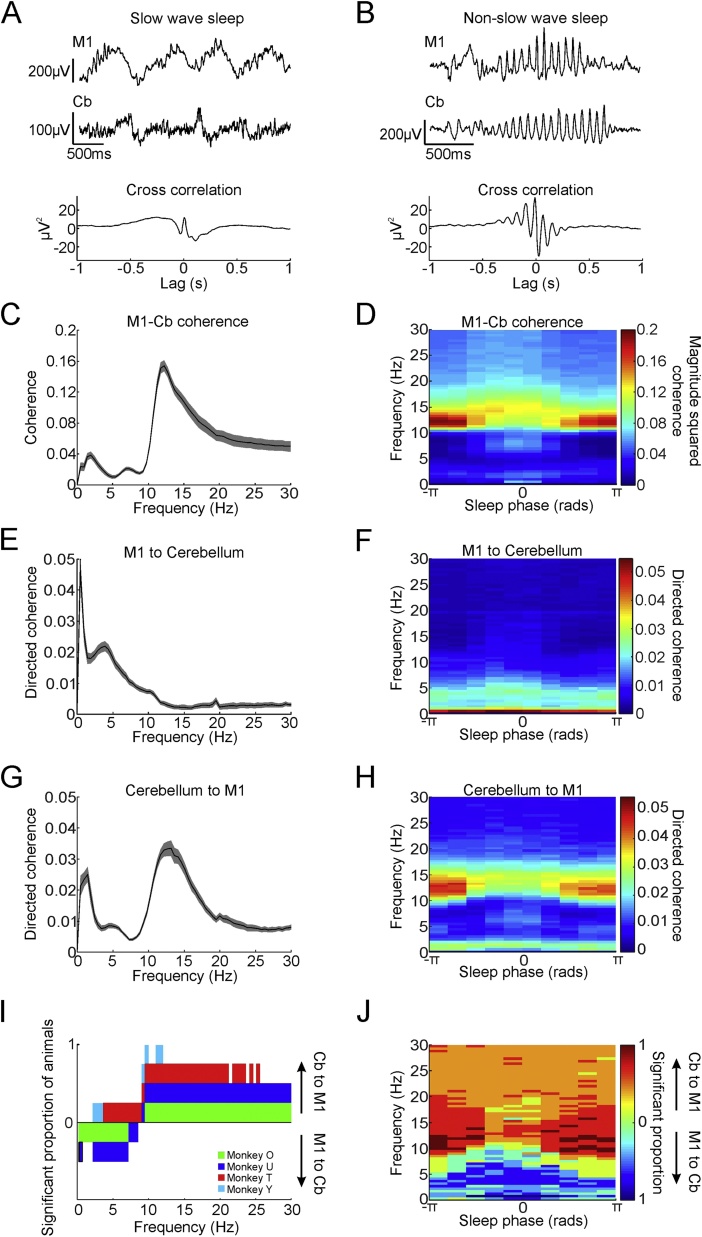
Functional connectivity between M1 and cerebellar LFPs. A. Example M1 and cerebellar LFP and their cross-correlation during SWS. B. Example M1 and cerebellar LFP and their cross-correlation during non-SWS. C,D. Mean magnitude-squared coherence between M1 and cerebellum for entire sleep duration and as a function of sleep phase. E,F. Mean directed coherence from M1 to cerebellum for whole sleep duration and as a function of sleep phase. G,H. Mean directed coherence from cerebellum to M1 for whole sleep duration and as a function of sleep phase. Shading indicates s.e.m. Data from all sessions in monkey U. Supplemental Fig. 4 shows corresponding plots for all animals. I,J. Proportion of animals with significantly different directed coherence for M1-to-cerebellum and cerebellum-to-M1 directions for whole sleep duration and as a function of sleep phase (paired *t*-test, P < 0.05). Values in the upper (lower) half of the plot represent M1-to-cerebellum directed coherence being significantly larger (smaller) than cerebellum-to-M1.

Given the correspondence between sleep rhythms in the neocortex and cerebellum, we next sought evidence for functional interactions between these structures. During SWS, cross-correlations between slow-wave activity in M1 and the cerebellum exhibited low-frequency oscillatory coupling (Fig. 2A). During non-SWS, correlated spindle-like rhythms were observed in both regions, evident as waxing and waning fast oscillations (Fig. 2B). We used frequency-domain coherence analysis to confirm the statistical significance of coupling between M1 and cerebellar LFPs in both delta and spindle bands (Fig. 2C). Across the whole night, the frequency of maximum coherence occurred in the spindle (9−16 Hz) range in 107/145 sessions, and the average (± s.e.m.) coherence across this range (0.088 ± 0.0056) exceeded the P = 0.05 significance in all 145 sessions. The frequency of maximum coherence occurred in the low/delta (0−4 Hz) range in only 21/145 sessions, although the average coherence across this range (0.056 ± 0.0045) nevertheless also exceeded the P = 0.05 significance in all sessions. Calculating coherence separately for different sleep phases confirmed that spindle-band coupling was predominant in non-SWS while slow-wave coupling was strongest in SWS (Fig. 2D).

To determine the directionality of cerebro-cerebellar interactions at different frequencies, we calculated directed coherence spectra. Interestingly, this revealed a clear directional dissociation between frequency bands. Slow-wave coherence during SWS was associated with greatest influence in the direction from the neocortex to cerebellum (Fig. 2E,G). By contrast, directed coherence at spindle frequencies in non-SWS was associated with a directionality from the cerebellum to the neocortex (Fig. 2F,H) consistently across all animals (Supp. Fig. 4). Fig. 2I shows frequencies for which directed coherence was significantly greater (two-tail paired *t*-test across sessions, P < 0.05) in one or other direction for all animals. A clear transition is seen between significant cerebro-cerebellar directionality at lower frequencies, and significant cerebello-cerebral directionality at higher frequencies. As an additional test, we calculated directed coherence on time-reversed LFP signals (Winker, Panknin et al. 2016). As expected, reversing the direction of time reversed the observed directionality of interactions at low and high frequencies (Supp. Fig. 5 A). Finally, Fig. 2J shows that instances of significant cerebro-cerebellar directionality occurred most often during SWS, while significant cerebello-cerebral directionality occurred most often during non-SWS.

### 3.3 M1 and cerebellar neurons exhibit phase-coupling at slow-wave and spindle-frequencies

**Fig. 3:**
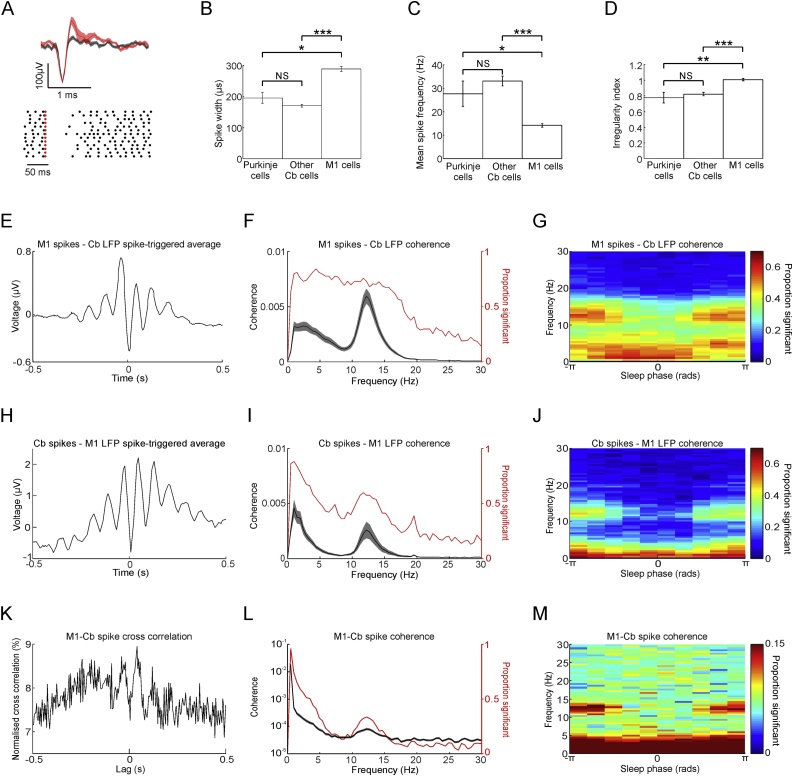
Functional connectivity between M1 and cerebellar neurons. A. *Top*: average waveform of 10 putative complex spikes (red) and simple spikes (black). *Bottom:* Raster of 10 simple spike trains aligned by complex spike. B-D. Bar charts comparing mean spiking properties (spike width, spike frequency, irregularity index) of identified cerebellar Purkinje cells, putative Purkinje cells and M1 neurons. P values calculated from Tukey-Kramer multiple comparisons tests (NS, *, ** and *** represents P > 0.05, P < 0.05, P < 0.01 and P < 0.001 respectively). E. Example spike-triggered average of cerebellar LFPs using spikes from an M1 neuron. F. Mean (±s.e.m.) magnitude-squared spike-LFP coherence between M1 neurons and cerebellar LFP (black), and the proportion of neuron-LFP pairs with significant coherence at each frequency (red). G. Proportion of all neuron-LFP pairs exhibiting significant coherence as a function of frequency and sleep phase. H-J. Same analysis as F-H, but between cerebellar spikes and M1 LFPs. K. Example cross-correlogram between an M1 and a cerebellar neuron. L. Mean (±s.e.m.) magnitude-squared spike-spike coherence between M1 and erebellar neuron pairs (black), and the proportion of neuron pairs with significant coherence at each frequency (red). M. Proportion of all neuron pairs exhibiting significant spike-spike coherence as a function of frequency and sleep phase.

We used differential LFP signals from electrode pairs located within the cerebellum to minimise volume conduction of field potentials from overlying neocortex. Nevertheless, to confirm coupling between the neocortex and cerebellum in sleep, we additionally sought evidence for correlations in simultaneous single-unit recordings. Note that, due to the low-impedance of the flexible microwires used for our chronic recordings (typically <0.5MΩ), definitive identification of Purkinje cell complex spike waveforms was not possible in all cases. However, in 11/113 cerebellar recordings we observed complex spikes followed by simple spike pauses characteristic of Purkinje cells (Fig. 3A). These positively-identified Purkinje cells did not significantly differ from the remaining cerebellar dataset in terms of peak-to-peak spike widths (Vigneswaran, Kraskov et al. 2011), which were similar to those previously reported for Purkinje cells (Bean, 2007). Additionally there were no significant differences in mean firing frequencies or irregularity index (P < 0.05, one-way ANOVA test with post-hoc pair-wise Tukey-Kramer multiple comparisons, Fig. 3B–D). Note that these metrics were capable of distinguishing between cerebellar and M1 neurons, with the latter exhibiting the wider waveforms, lower firing rates and higher irregularity that are characteristic of neocortical pyramidal neurons (Fig. 3B–D). By contrast, Purkinje cells are known to fire simple spikes steadily at high frequencies with fast repolarization (Bean, 2007) suggesting our cerebellar sampling is likely biased towards these neurons, although we do not discount the possibility of other cerebellar cell types being present in the dataset.

We first compiled spike-triggered averages of LFPs recorded in M1 or the cerebellum, triggered by neurons in the other area, to reveal oscillatory coupling across areas (Fig. 3E, H). Since the statistical significance of oscillatory coupling is more readily assessed in the frequency domain, we also calculated spike-LFP coherence spectra. Clear peaks around spindle band frequencies were observed in the average coherence between all M1 spikes to cerebellar LFPs (Fig. 3F), and all cerebellar spikes to M1 LFPs (Fig. 3I). In this band, mean coherence reached statistical significance (P < 0.05) for 207/265 individual M1 neurons and 59/113 individual cerebellar neurons. As before, spindle-frequency coupling was associated primarily with non-SWS phases (Fig. 3G, J).

Next, we examined cross-correlation histograms for all simultaneously-recorded pairs of M1 and cerebellar neurons. Evidence for cortico-cerebellar spike-spike interactions have previously proved challenging to demonstrate in awake animals (Holdefer, Miller et al. 2000), but we were able to resolve weak features in the cross-correlograms between some pairs of neurons in our sleep datasets (example in Fig. 6K). Spindle-band coupling was revealed more clearly in average spike-spike coherence spectra, with significant (P < 0.05) coherence observed for 90/493 cell pairs (Fig. 3L) and associated predominantly with non-SWS phases (Fig. 3M). Thus we conclude that the coupling observed between cerebellar and M1 LFPs reflects interactions between neurons in the vicinity of the recording electrodes and not volume conduction from distant sources.

### 3.4 Communication through the cerebello-thalamo-cortical pathway at spindle frequencies

**Fig. 4:**
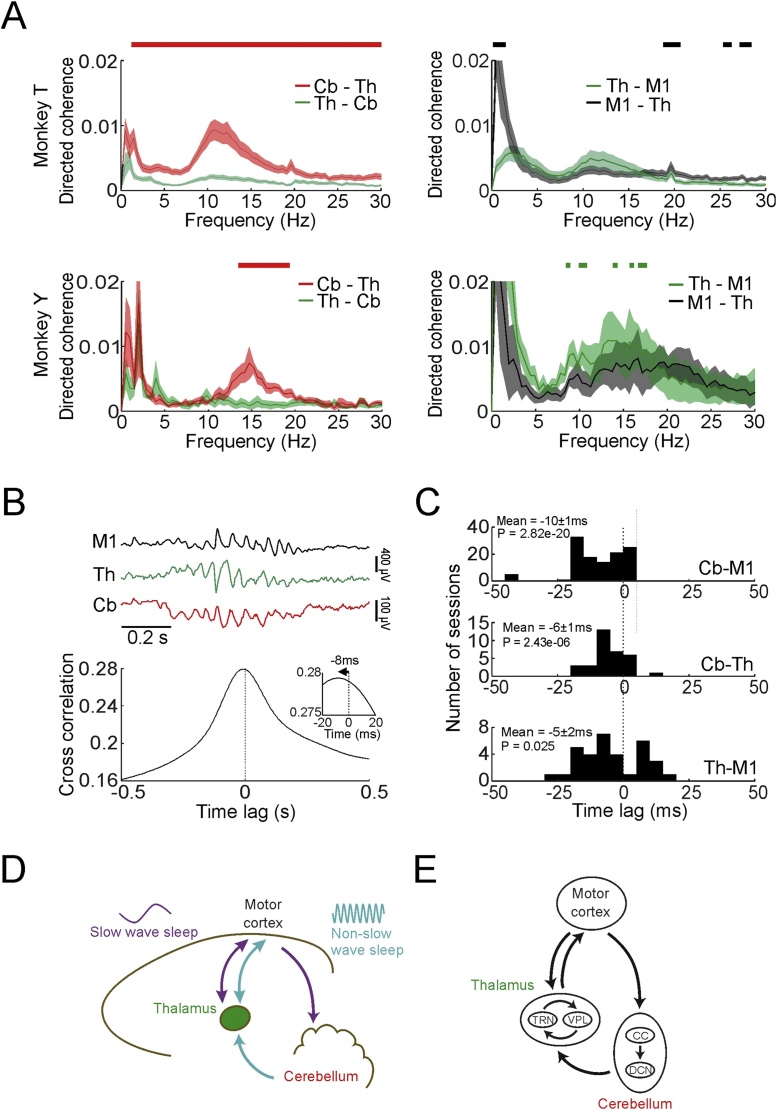
Spindle frequency signals are transmitted from the cerebellum to M1 via the thalamus. A. Directed coherence between cerebellum and thalamus, and thalamus and M1, for two animals. Frequencies with significant directionality indicated by coloured lines (P < 0.05, paired *t*-test across sessions). B. *Top:* Example of simultaneously recorded spindle-like events from M1, thalamus (Th) and cerebellum (Cb). *Bottom:* Cross-correlation between spindle-band amplitude envelopes in M1 and cerebellum for an example session. The offset cross-correlation peak shows the cerebellum leading M1. C. Histograms of cross-correlation peak times for pair-wise comparisons between areas across all sessions. P values are for one-sample *t*-test. D. Schematic summary of the main direction and frequency of communication during SWS and non-SWS. E. Schematic summarizing the known anatomical connectivity in the cerebello-thalamo-cortical circuit (CC, DCN, TRN and VPL represent cerebellar cortex, deep cerebellar nuclei, thalamic reticular nucleus and ventroposterior-lateral thalamus respectively).

A putative pathway by which oscillatory activity could be relayed from the cerebellum to the neocortex is via the disynaptic connection through the thalamus (Sasaki, Kawaguchi et al. 1976). Therefore, in two animal we placed electrodes in the VPL nucleus of the thalamus, which receives inputs from the deep cerebellar nuclei and projects to motor cortex (Kalil, 1981). For both of animals, there was significantly greater directed coherence from cerebellum-to-thalamus than in the reverse direction (Fig. 4A, left; P < 0.05, paired *t*-test across sessions). This was true even when using the differential signal from two adjacent electrodes within the VPL, separated by 1.2 mm to provide an extremely localised VPL recording (Supp. Fig. 5B). Between the thalamus and M1, directed coherence was also observed at spindle frequencies. Although this was greatest in the direction from M1 to thalamus, this difference only reached statistical significance in one animal (Fig. 4A, right).

We performed a second analysis to compare the relative timing of spindle-band activity in the three brain regions. We first extracted the amplitude envelope of 9−16 Hz oscillations in each area using a Hilbert transform, and applied pairwise cross-correlations to identify the time lags associated with peak correlation. Fig. 4B shows example spindle events and cross-correlations for a single session, and Fig. 4C summarises peak correlation lags for all sessions. Peak correlation between M1 and cerebellar spindle-band activity occurred with cerebellum leading M1 by 10 ± 1 ms (mean ± s.e.m), and this was significantly different from zero (n = 121 sessions, P = 3 × 10^−20^, *t*-test, Fig. 4B). This method also showed cerebellar spindle activity leading thalamus by 5 ± 1 ms, and thalamus leading M1 by 6 ± 2 ms, both significantly different from zero (n = 34 sessions, P = 2 × 10^-6^ and P = 0.025 respectively, Fig. 4C). These values are consistent with known conduction times from the cerebellum to M1 in monkeys (Holdefer, Miller et al. 2000), and the fact that thalamic spindles precede neocortical spindles in humans (Mak-McCully, Rolland et al. 2017). A schematic summary of our directed coherence results is shown in Fig. 4D, consistent with the known anatomy of the cerebello-thalamo-neocortical pathway (Fig. 4E).

### 3.5 Communication through the cerebello-thalamo-cortical pathway during identified sleep spindle events

**Fig. 5:**
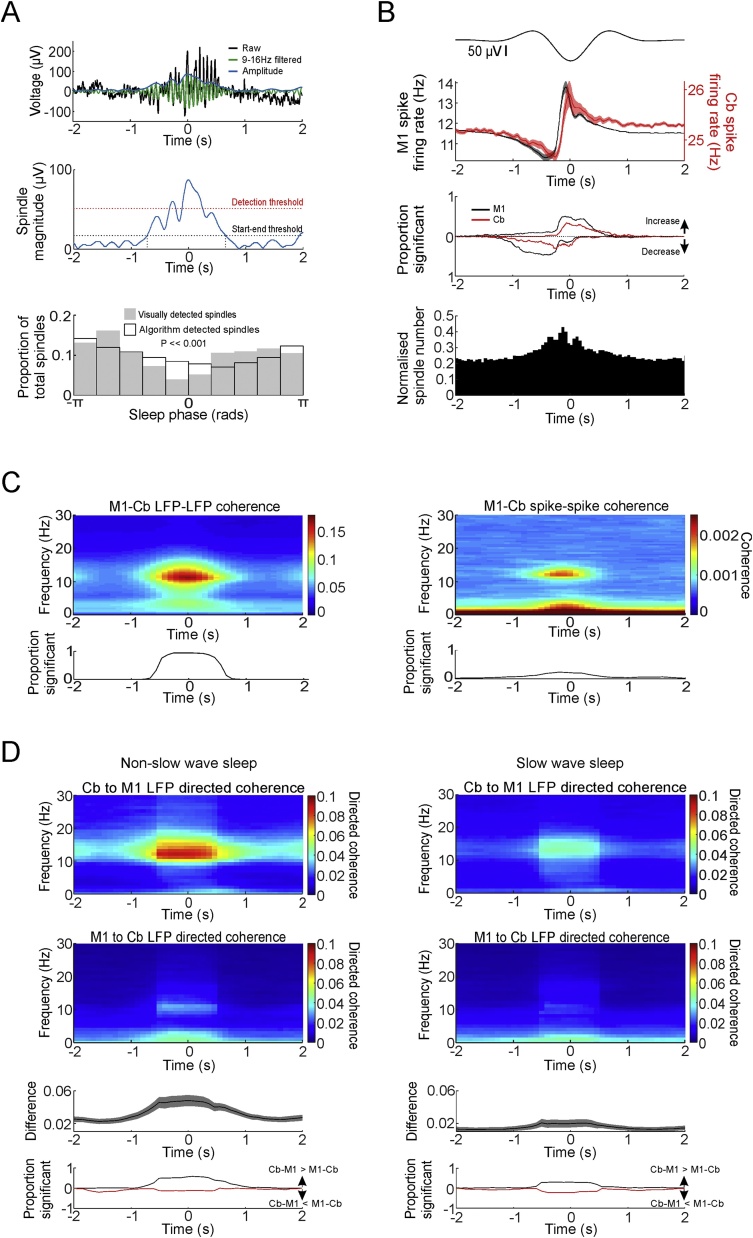
Coherence and directed coherence aligned by identified M1 sleep spindles. A. *Top:* Example of a sleep spindles in M1 detected by the automated algorithm. Green trace plots 9-16 Hz filtered signal. Blue trace plots instantaneous amplitude of filtered spindle. *Middle:* Spindle magnitude with detection and start-end thresholds. *Bottom:* Distribution of automatically detected spindles against sleep phase averaged across all sessions (white bars), and distribution of manually detected spindles (grey bars). P value derived from Rayleigh’s test of circular non-uniformity. B. *Top:* Mean M1 LFPs aligned by identified slow wave negative peaks (up-states). *Middle:* Mean firing rate of M1 (black) and cerebellar (red) neurons aligned to up-states. Also shown is proportion of M1 and cerebellar neurons with significant firing rate modulation (3 SD above/below baseline assessed between −3 to −2 s and 2 to 3 s relative to up-state event). *Bottom:* Distribution of identified spindles aligned by up-states (50 ms binwidth). C. *Top:* mean coherence between M1 and cerebellar LFPs and spikes aligned to peak spindle amplitude. *Bottom:* proportion of significant sessions with significant coherence modulation in the spindle band. D. Connectivity associated with identified spindles in non-slow-wave sleep (*left*) and slow-wave sleep (*right*). Upper plots show directed coherence from cerebellum to M1 and M1 to cerebellum aligned by peak spindle amplitude for nSWS and SWS. Lower plots show difference between cerebellum-to-M1 and M1-to-cerebellum directed coherence in the spindle band, and proportion of sessions with positive/negative modulation of this difference compared to baseline ±3 SD. Shading indicates s.e.m.

The human sleep EEG literature distinguishes spindles from sleep alpha rhythms which have a lower frequency and occipital topography (Cantero, Atienza et al. 2002). In this regard, the oscillations we observed in locally-referenced M1 LFPs appear consistent with sleep spindles, and have a similar frequency to previous reports of spindles in monkeys (Takeuchi, Murai et al. 2016; Sritharan, Contreras-Hernandez et al. 2020). A further signature of spindles is their waxing and waning amplitude, which is maximal during up-states of the cortical slow oscillation (Contreras and Steriade, 1995). In order to verify that the spindle-frequency coupling between M1 and cerebellum in our dataset was associated with these characteristics, we used a previously published method (Andrillon et al., 2011) to automatically identify spindle events from the LFP based on their temporal dynamics (Fig. 5A). Before applying this algorithm, we first removed putative periods of REM sleep and arousals by applying a threshold to high-gamma activity (see Section 2.7 and Supp. Fig. 2).

Automatically identified spindles were most prevalent during non-slow-wave sleep (Fig. 5A, unfilled bars), occurring at an average (± s.e.m.) rate of 1.58 ± 0.03, 1.03 ± 0.04 and 1.86 ± 0.04 per minute during for monkeys O, U and T respectively. By contrast the rate of spindles in slow-wave sleep was 1.01 ± 0.03, 0.61 ± 0.03 and 1.40 ± 0.06 per minute respectively, and these differences were statistically significant (P = 5 × 10^−14^, 5 × 10^-28^, 7 × 10^-07^, paired *t*-test over sessions). For one example session (from monkey O), we confirmed the automatic method by visually identifying spindles to again reveal the same pattern (Fig. 5A, grey bars).

To examine how these identified spindles were related to the cortical slow oscillation, we used a previously published method (Nir, Staba et al. 2011) to identify putative up-states from negative peaks in the depth LFP. These events were associated with elevated firing rates of M1 neurons, and were preceded by relative suppression (Fig. 5B), similar to previous findings in humans (Nir, Staba et al. 2011). Interestingly, cerebellar firing rates exhibited a similar pattern but the down- and up-states lagged those in the neocortex by about 100 ms, consistent with a neocortical origin of the slow oscillation (Rowland, Goldberg et al. 2010). Identified spindles were most prevalent around the time of the up-state, consistent with previous findings that spindles tend to occur after down- to up-state transitions (Molle, Marshall et al. 2002; Mak-McCully, Rolland et al. 2017).

Next we used a sliding window approach to assess coupling between M1 and cerebellum during identified spindle events. Coherence in the spindle frequency range was greatest during the spindle for both LFPs and spikes (Fig. 5C). Similarly, directed coherence between M1 and cerebellar LFPs showed a prominent peak, which was greatest in the cerebellum-to-M1 direction (Fig. 5D). Note also that directed coherence from cerebellum to M1 was lower for spindles occurring in SWS compared with spindles in non-SWS. Thus the modulation of spindle-band connectivity through the sleep cycle reflected the combination of two factors. First, there were fewer spindles in SWS and, second, these spindles were associated with weaker connectivity from the cerebellum to neocortex.

**Fig. 6:**
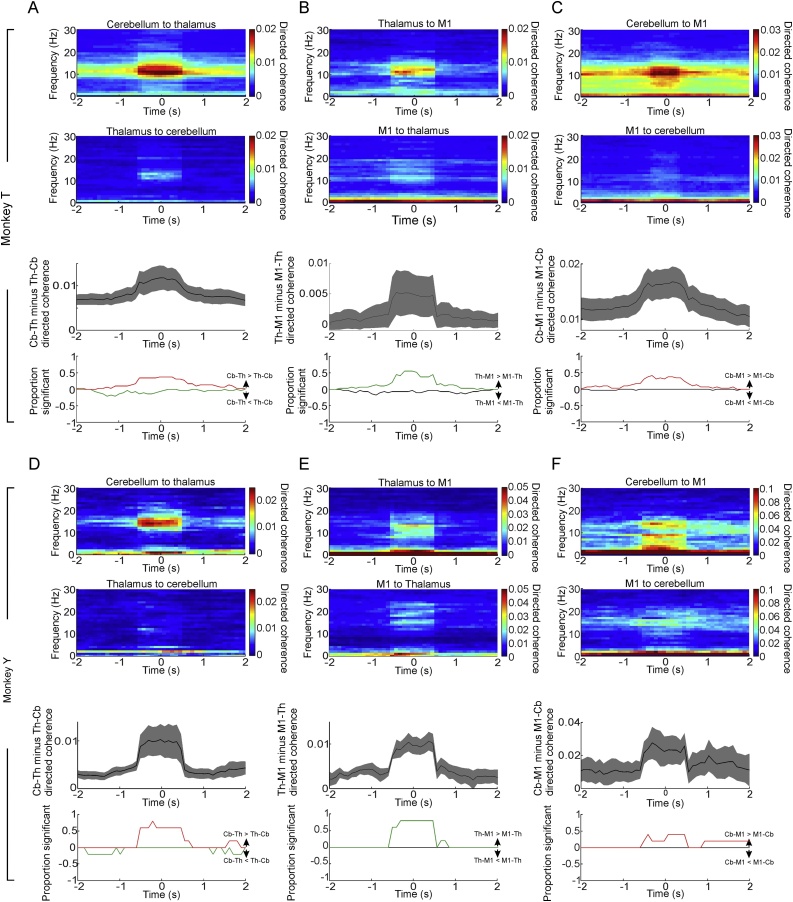
Directed coherence aligned by identified thalamic spindles. A. Upper plots show directed coherence from cerebellum to thalamus and from thalamus to cerebellum, aligned by peak thalamic spindle amplitude. Lower plots show difference between cerebellum-to-thalamus and thalamus-to-cerebellum directed coherence in the spindle band, and proportion of sessions with positive/negative modulation of this difference compared to baseline ±3 SD. Data from Monkey T. Shading indicates s.e.m. B. Same but for thalamus and M1. C. Same but for cerebellum and M1.D-F. Same but for Monkey Y.

For monkeys T and Y we additionally applied the algorithm of Andrillon et al. (2011) to identify spindles in thalamic recordings. Directed coherence aligned to these thalamic spindles showed a clear directionality from the cerebellum to the thalamus (Fig. 6A,D), from the thalamus to M1 (Fig. 6B,E), as well as from cerebellum to M1 (Fig. 6C,F).

### 3.6 Cerebro-cerebellar connectivity during different sleep stages

**Fig. 7:**
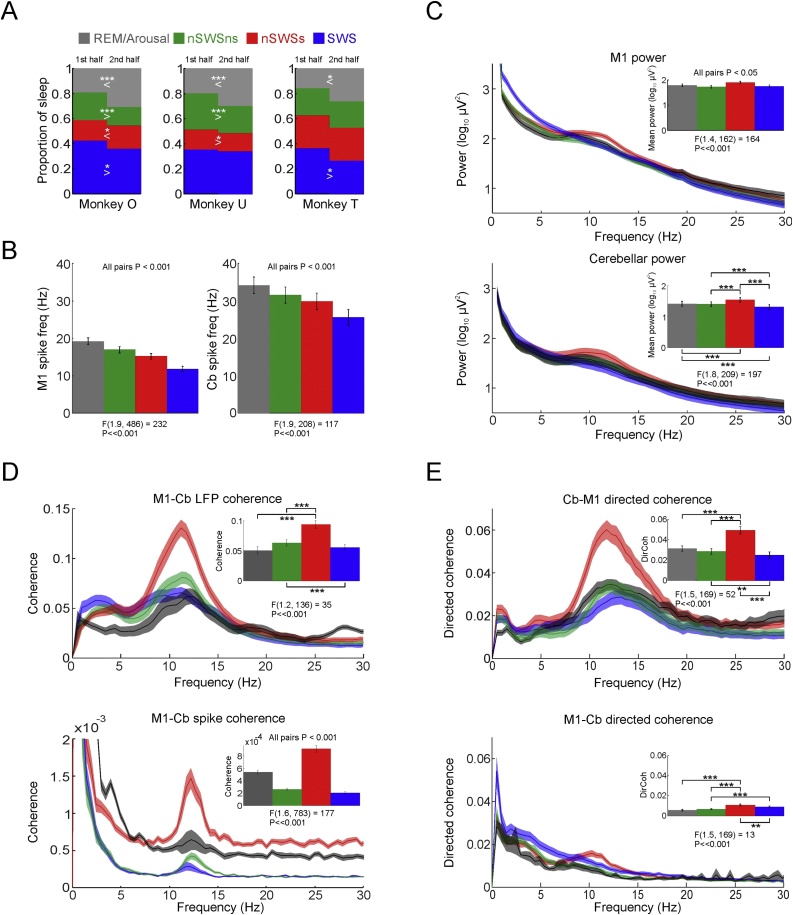
Coherence and directed coherence analysis for discrete sleep stages. A. Proportion of total sleep time spent in each of REM/arousal, slow-wave sleep (SWS), non-slow-wave sleep with spindles (nSWSs) and non-slow-wave sleep without spindles (nSWSns) for monkeys O, U and T in the first and second halves of the night. B. Mean M1 and cerebellar spike firing rate for each sleep stage. C. Mean LFP power for M1 and cerebellum for each sleep state. D. *Top:* Mean cerebellum-M1 coherence for each sleep state. *Bottom:* Mean M1-to-cerebellar spike-to-spike coherence for each sleep state. E. Mean cerebellum-to-M1 (*top*) and M1-to-cerebellum (*bottom*) directed coherence for each sleep state. Inset bar charts show average across the spindle band. Data from all sessions in all animals. F ratio and P values obtained from repeated-measure ANOVA (with Greenouse-Geisser correction) across sessions. *, ** and *** indicate P < 0.05, 0.01 and 0.001 respectively (post-hoc paired *t*-test with Bonferroni correction).

We combined our circular sleep phase measure, automated spindle identification, and high-gamma band criteria (see Sections 2.6–2.8) to classify 30s-long windows as REM/arousal, slow-wave sleep (SWS), non-slow wave sleep without spindles (nSWSns) or non-slow wave sleep with spindles (nSWSs, Fig. 7A). Although not identical to standard AASM criteria, the latter two states were qualitatively similar to stages N1 and N2 respectively. The second half of the night was associated with a greater proportion of REM/arousal and reduced SWS compared to the first half of the night (Fig. 7A), in keeping with a homeostatic decrease in sleep pressure (Vyazovskiy, Riedner et al. 2007). Neuronal firing rates in both M1 and cerebellum were highest for REM/arousal states, and were progressively reduced through nSWSns, nSWSs to SWS (Fig. 7B), consistent with increasing sleep depth. LFP spectra during SWS (Fig. 7C) showed pronounced low-frequency power, while nSWSs epochs were characterised by reduced delta and a spindle-frequency peak. We then repeated our coherence and directed coherence analyses separately for each of these four sleep states. This confirmed that LFP-LFP coherence (Fig. 7D, top), spike-spike coherence (Fig. 7D, bottom) and directed coherence from cerebellum to M1 (Fig. 7E) were all significantly modulated by sleep state (P < 0.05, one-way ANOVA across sessions) and were greatest during non-slow wave sleep with spindles.

### 3.7 Phase-dissociation of M1 and cerebellar sleep spindles suggests coupled oscillators

These directed coherence results are surprising because spindles are generally thought to arise from the thalamus, in part due to well-established oscillatory characteristics of TRN neurons which inhibit thalamo-cortical cells (Crabtree, 2018). However, *in vivo* the thalamus is likely coupled with other oscillatory networks within the cerebello-thalamo-cortical system, and standard directed coherence metrics do not distinguish between a single oscillation propagating from one site to another (or a single oscillator projecting to both sites with different lags) from a system of multiple, coupled oscillators. We speculated that the presence of such coupled oscillators might be revealed by systematic variations in their relative phase (Appleby, 2014). A well-known example of this is the continual back-and-forth transfer of energy between a pair of coupled pendulums, such that alternating periods of phase lead and lag are associated with a decreasing/increasing amplitude of oscillation in the driving/driven pendulum.

**Fig. 8:**
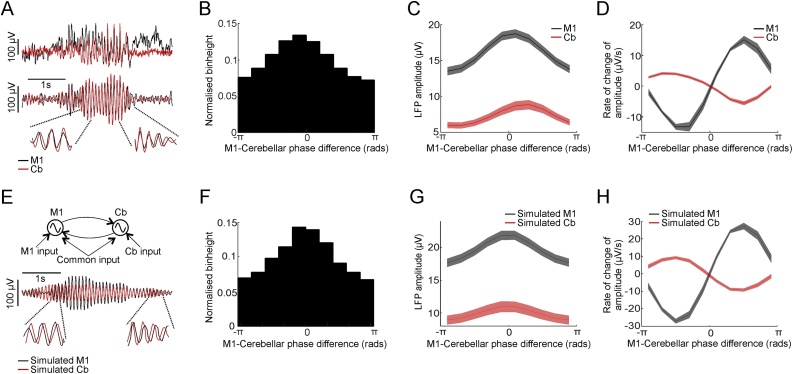
Phase relationship between M1 and Cerebellar spindles. A. *Top:* Example M1 (red) and cerebellar (black) spindles. *Bottom:* The same signals filtered between 7-15 Hz. B. Histogram of instantaneous phase differences between spindle-band M1 and cerebellar signals for example session. C. Mean amplitude of M1 and cerebellar spindle oscillations against their instantaneous phase difference (relative to preferred phase for each session). Shading indicates s.e.m. over all sessions with monkey U. D. Plot of mean amplitude derivative for M1 and cerebellar spindle oscillations against their instantaneous phase difference. E. Schematic of bidirectional coupling between spindle oscillators (*top*) and example model output (*bottom*). F—H. Same as BD— but for simulated M1 and cerebellar signals fit to each session in Monkey U. See Supplemental Fig. 6 for phase and phase derivative relations of all animals.

Inspection of the raw LFPs (Fig. 8A *top*) suggested that the M1-cerebellar phase-difference often changed through the time course of spindles. To examine this further, we used the Hilbert transform of the band-pass filtered LFP (7−15 Hz to encompass width of the directed coherence peaks observed; Fig. 4A *bottom*) to calculate the amplitude envelopes and instantaneous phase difference between M1 and cerebellar oscillations (see Section 2.11). We performed this analysis for the three animals for which we had the most sessions (O, U and T). Fig. 8B shows a histogram of spindle phase-difference during an entire night, divided into ten bins spanning –π to π. While instances of all possible phase-differences occurred throughout the recording, the presence of a histogram peak indicates a tendency for these oscillations to be locked to a preferred relative phase. Significant phase coupling (Rayleigh test for circular non-uniformity over samples; P < 0.05) was observed in 100 % of sessions, and the preferred phase was consistent for different sessions with the same animal (Rayleigh test for circular non-uniformity over sessions; Monkey O: P = 5 × 10^−19^, U: P = 1 × 10^-4^, T: P = 2 × 10^-6^). Across animals, the preferred phase varied, as is to be expected since the polarity of LFP recordings will depend on the precise positioning of electrodes.

Next, we plotted the average instantaneous amplitude of M1 and cerebellar spindle oscillations through the whole night for all time-points that were associated with each phase difference (relative to the preferred phase of coupling for that session, Fig. 8C). Amplitudes in both areas were maximal close to the preferred phase, as might be expected since the phase measurements of weaker signals will be more affected by noise. Finally, we plotted the mean time-derivative of amplitude envelopes associated with each relative phase (Fig. 8D). Interestingly, this analysis revealed that increases/decreases in the amplitude of M1 and cerebellar spindles were associated with distinct relative phases. This appears inconsistent with a single oscillation propagating from one area to another (or a single oscillator driving both areas), as these would exhibit a consistent relative phase irrespective of amplitude changes. Instead, a phase difference that fluctuates systematically with changing amplitudes is reminiscent of the behaviour of the coupled pendulums described above. Therefore to explore this intuition further we turned to linear dynamical systems theory.

### 3.8 A dynamical system model of coupled M1-Cerebellum oscillators

To demonstrate that the systematic relationship between spindle phase-difference and amplitude changes could be explained by the interaction of two oscillatory networks, we simulated a simple linear dynamical system comprising two coupled oscillators driven by white noise (Fig. 8E; *top*). The system dynamics were determined from a linear fit of the 7−15 Hz band-pass filtered LFP data (after applying the Hilbert transform), and the correlation structure of the noise input was matched to the covariance of the residuals (see Section 2.11). Conceptually, this approach is similar to the autoregressive modelling that underlies the calculation of directed coherence, except that by focussing on a narrower frequency band we can greatly reduce the number of model parameters. Simulated LFPs (Fig. 8E; *bottom*) generated by this simple dynamical system under white noise input exhibited similar phase-amplitude relationships to the real data (Fig. 8F–H). Indeed, although the phase-amplitude relationships varied across animals, the same model could capture all of the observed patterns (Supp. Fig. 6A,B) with appropriate choice of only 12 free parameters (2 × 2 complex dynamics matrix, two real noise variances and a complex covariance; see Section 2.11). Thus, although the true circuitry doubtless contains more complexity, our model of two coupled linear oscillators was *sufficient* to explain the observed relationships between phase-difference and amplitude/amplitude-derivatives.

**Fig. 9:**
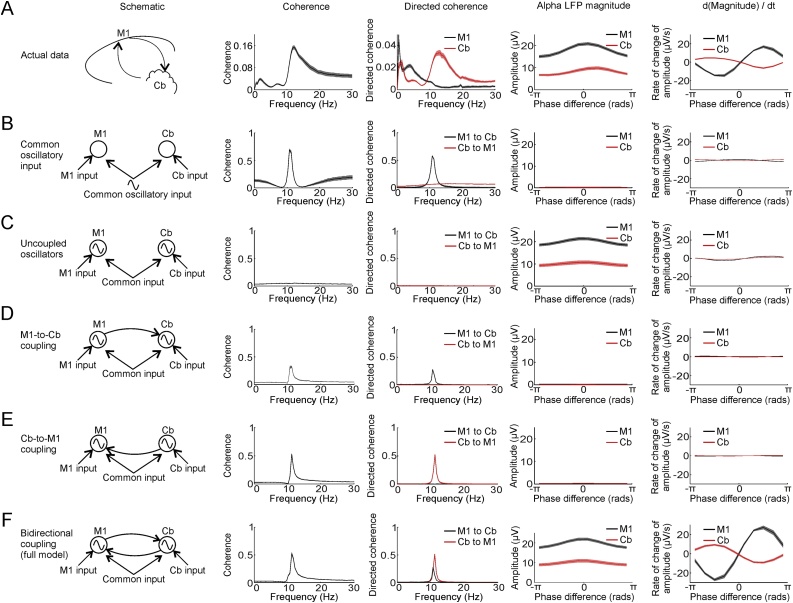
Comparison of full and reduced dynamical system models to actual data. A. Experimental data from Monkey U. From left to right: schematic of neuronal circuit, mean magnitude-squared coherence between M1 and cerebellar LFPs, mean directed coherence for M1-to-cerebellum and cerebellum-to-M1, mean amplitude of M1 and cerebellar spindles against their phase difference, mean amplitude derivative of M1 and cerebellar spindles against their phase difference. B-F. Similar to A but for simulated data from each model indicated by the schematic to the left. Shaded regions represent s.e.m. over all sessions in Monkey U. See Supplemental Fig. 6 and Supplemental Table 1 for model comparisons in all animals.

Next, we considered whether all components of our coupled oscillator model were *necessary* to explain our data by fitting five reduced models using the same approach (Fig. 9; see Section 2.11). In brief, these models either assumed a single common source for oscillations in M1 and cerebellum (incorporating different phase delays), or ignored one or both directions of coupling. Two analyses demonstrated that none of the reduced models fully captured our experimental data. First, in cross-validation on data not used to fit parameters (to safeguard against over-fitting), performance of the reduced models (as measured by the mean-squared residual) was significantly worse (two-tail paired *t*-test over sessions, P < 0.05) for all but one comparison across the three animals (Supp. Table 1). Second, none of the reduced models could explain the systematic relationship between relative phase and amplitude changes we observed for M1 and cerebellar spindles-band oscillations (Fig. 8). In particular, the similarity between the actual and simulated phase-difference vs. amplitude-derivative curves (as measured by linear correlation) was significantly worse (two-tail paired *t*-test over sessions, P < 0.05) for all reduced models compared to the full model in all animals (Supp. Fig. 6C). Thus, we conclude that a dynamical system comprising two coupled oscillators is both necessary and sufficient to explain the systematic fluctuation in the M1-cerebellar phase-difference with changes in spindle-band amplitudes.

Note that despite the bidirectional coupling in our dynamical system model, the direction of greatest causal influence in the spindle band (as quantified by directed coherence) was nevertheless from the cerebellum to M1, in agreement with our earlier results. This is because directed coherence reflects the combined effect of coupling coefficients together with the origin of the oscillations (innovation noise in the model). It should also be borne in mind when interpreting our data that oscillations observed in one area may nevertheless originate from networks upstream of that site. Therefore, taken together our analyses demonstrate that (i) a network that incorporates (or projects to) the cerebellum generates spindle-frequency oscillations in the cerebellum, (ii) the cerebellum is coupled bi-directionally with a second oscillatory network that incorporates (or projects to) M1, e.g. the thalamus, and (iii) the predominant direction of communication at spindle frequencies is from the cerebellum to the thalamus and M1.

## 4 Discussion

We have shown that the cerebellum is an active participant of sleep, exhibiting sleep cycles characterised by fluctuating firing patterns and reciprocal fast/slow oscillations that bear a remarkable similarity to the neocortex. Functional connectivity between the two brain areas was seen at population and single-unit levels, while causality measures revealed an unexpected reversal in the direction of information flow at the different frequencies that occur through the sleep cycle. At low frequencies associated with slow-wave sleep, the prevailing directionality was from the neocortex to the cerebellum, as has also been described in ketamine-anesthetised rats (Rowland, Goldberg et al. 2010). By contrast, during identified spindle events that occurred most often in non-SWS, the prevailing directionality was from cerebellum to the thalamus and neocortex, similar to that seen during awake behaviour (Watson, Becker et al. 2014).

Classically, spindles are believed to originate in the thalamus, evidenced both by the intrinsic rhythmicity of the thalamic reticular nucleus (TRN) and the disappearance of neocortical spindles after surgical disconnection from the TRN (Steriade, Deschenes et al. 1985; Steriade, Domich et al. 1987; von Krosigk et al., 1993; Luthi, 2014). However, given the absence of direct thalamo-cerebellar projections, the principle route by which spindles from the TRN could influence the cerebellum is via the neocortex (Kelly and Strick, 2003). Our directed coherence analysis instead revealed signals at spindle frequencies and associated with identified spindle events propagating from the cerebellum, through the thalamus and into motor cortex. Moreover, we observed a systematic variation of the phase-difference between neocortical and cerebellar spindles associated with the waxing and waning of spindle amplitudes. Linear dynamical systems analysis showed that these observations were incompatible with the classical model of a single source for spindle oscillations located in the thalamus. Instead, we demonstrated that a model comprising two coupled oscillators was both necessary and sufficient to explain the observed cerebro-cerebellar interactions. While one of these oscillators likely involves the TRN, the other must either be intrinsic to or upstream of the cerebellum (Moises, Waterhouse et al. 1983; D’Angelo et al., 2009; Courtemanche, Robinson et al. 2013). Bidirectional coupling between these oscillators could be mediated by the known architecture of cerebellar-thalamo-cortical loops (Kelly and Strick, 2003). While the second oscillator could in principle be located within a separate region of the TRN projecting via a different route to the cerebellar cortex, we consider this unlikely based on the anatomical and functional segregation of these cerebellar-thalamo-cortical loops (Strick, Dum et al. 2009).

Note that our hypothesis is compatible with previous observations that neocortical spindles depend on the integrity of the thalamus (since the cerebellar nucleus projects to the thalamus) and the intrinsic rhythmicity of the isolated TRN. However, unlike the classical model of a single spindle source within the thalamus, it can also explain directed coherence from the cerebellum to the thalamus and neocortex as well as the variation of relative phase observed in our dataset. It is increasingly recognised that, rather than a single global rhythm, neocortical sleep spindles are heterogeneous, with fast and slow frequencies (Molle, Bergmann et al. 2011), global and local patterns of synchronisation (Dehghani, Cash et al. 2010; Andrillon et al., 2011; Bastuji, Lamouroux et al. 2020) and different laminar profiles (Hagler, Ulbert et al. 2018). The extent to which distinct or coupled oscillatory networks account for this heterogeneity remains an open question, but our results highlight the importance of also considering cerebellar inputs to the thalamus and their influence on thalamo-cortical oscillations (Gornati, Schafer et al. 2018). It would be interesting in future to confirm our functional connectivity results by lesion or inactivation of cerebellar structures during sleep, although such studies represent a considerable technical challenge in non-human primates. However, a cerebellar contribution to sleep spindles is consistent with the reduction in neocortical spindle density observed in humans with conditions linked to cerebellar atrophy, including spinocerebellar ataxias (Seshagiri, Botta et al. 2018), schizophrenia (Manoach, Thakkar et al. 2010) and autism (Farmer, Chilakamarri et al. 2018).

Similar to Mak-McCully, Rolland et al. (2017), the spindles in our study predominantly occurred after neocortical down-states. They proposed a mechanism whereby the neocortical down-state causes a thalamic down-state, and the resulting hyperpolarisation makes available intrinsic pace-maker currents to support spindle oscillations that then propagate back to the neocortex. Our data shows that the neocortical down-state also produces a subsequent reduction of firing in the cerebellum and it is interesting to speculate whether hyperpolarisation-activated pace-maker currents may also be involved in the generation of cerebellar spindles. Certainly, there is evidence for *I_h_* currents in Purkinje cells (Williams, Christensen et al. 2002) and in the deep cerebellar nuclei (Aizenman and Linden, 1999), and it has been proposed that synchronised oscillations in the inferior olive are controlled by similar intrinsic mechanisms to those operating in the TRN (Bal and McCormick, 1997). Thus, disfacilitation following neocortical down-states could be a common mechanism to support spindle generation in both the thalamus and cerebellum, but this hypothesis requires further experimental support.

Sleep spindles are often attributed a role in transferring episodic memories from the hippocampus for long-term consolidation in the neocortex (Diekelmann and Born, 2010; Staresina, Bergmann et al. 2015; Latchoumane, Ngo et al. 2017). However, spindle density, as well as time spent in N2 sleep, is also known to be predictive of off-line improvements in procedural tasks (Nishida and Walker, 2007; Diekelmann and Born, 2010; Fogel, Albouy et al. 2017). Spindles act as a thalamic gate to sensory information in sleep (Luthi, 2014), but our results suggest that spindle oscillations may afford the cerebellum preferential access to the neocortex during this period of enhanced synaptic plasticity (Niethard, Ngo et al. 2018). The cerebellum is thought to represent forward models that predict the sensory consequences of actions (Bastian, 2006), and we speculate that access to such predictive models could allow off-line practice and optimisation of motor skills during sleep.

## Funding

This work was supported by the Wellcome Trust (106149) and the 10.13039/501100000266Engineering and Physical Sciences Research Council (H051570).

## Data and code availability

Data and computer codes used in this study are available upon request to the first author. Example data is deposited in figshare dataset: https://doi.org/10.6084/m9.figshare.13200395.v1

## Author contributions

WX and AJ designed and carried out the experiments. WX, AJ and AKC analysed the data. FDC designed, manufactured and implemented the wearable recording device. WX and AJ wrote the manuscript, with contributions from all authors.

## Declaration of Competing Interest

The authors report no declarations of interest.
