# Supplementary material for: Communication from the cerebellum to the neocortex during sleep spindles
Source: Prog Neurobiol. 2021 Apr;199:101940. doi: 10.1016/j.pneurobio.2020.101940 (PMC7938225; doi:10.1016/j.pneurobio.2020.101940)
Supplement: Supplementary file 1 [file mmc1.docx]

**APPENDIX**


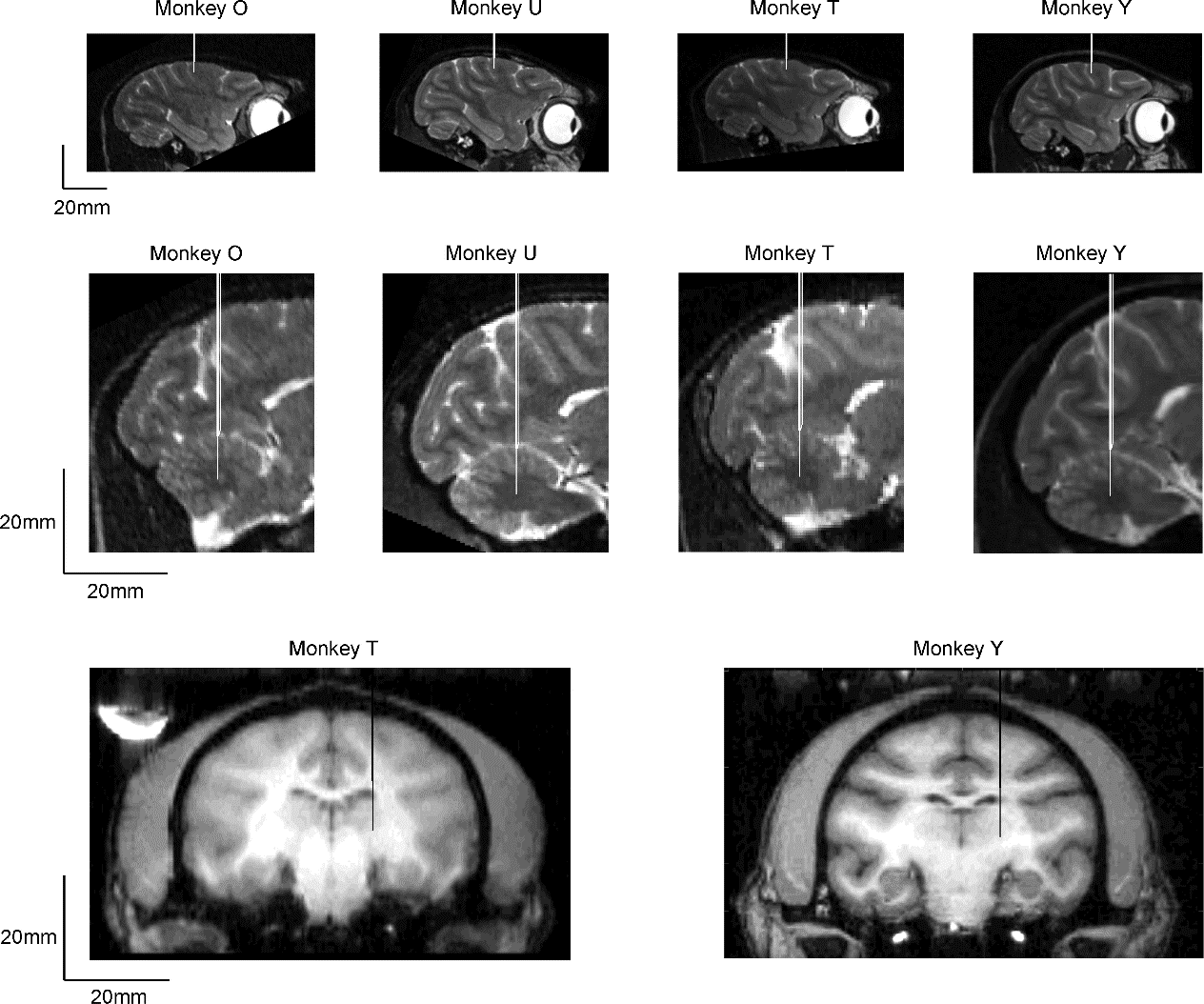


**Supplemental Figure 1.** MRI of all four animals with indicated M1 LMA, cerebellar LMA and guide tube, and thalamus LMA placement.


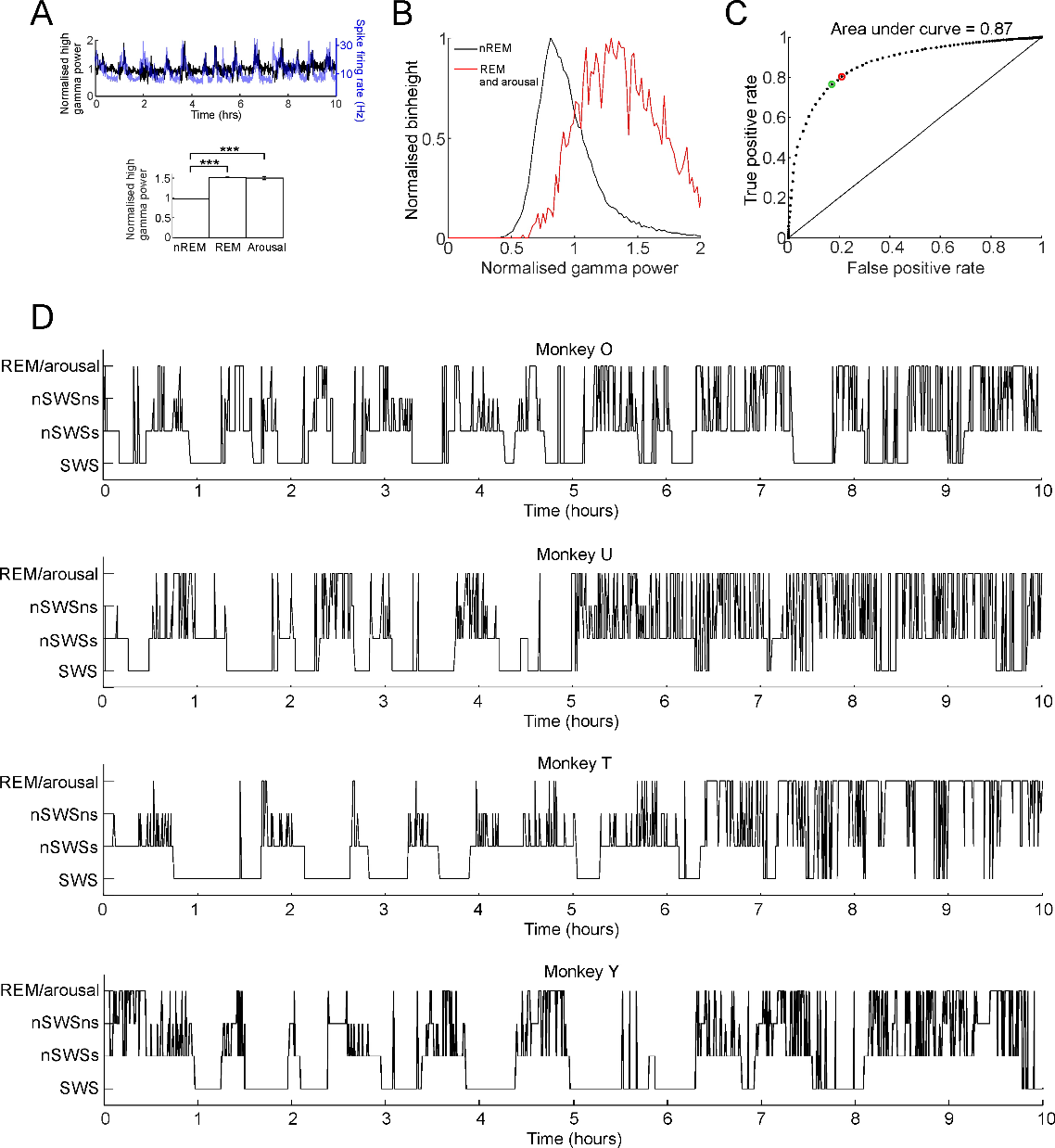


**Supplemental Figure 2. Identification of REM and arousal periods from high-gamma activity. A.** Example of M1 spike firing rate and broadband high-gamma power during entire sleep duration (*top*), and mean high-gamma power for non-REM, REM and arousal windows (*bottom*). High-gamma power was defined as mean power for 50-125 Hz over 30s-long windows, normalised by the average across the entire night. Data from 33 sessions in Monkey U. *** represents P < 0.001, one-way ANOVA with Tukey-Kramer correction). **B.** Distribution of normalised high-gamma power for identified nREM and REM/arousal windows. **C.** Receiver-operator curve for different discrimination thresholds. Red point indicates intersection with negative diagonal as well as point closest to top left corner. Green point indicates maximal Youden’s index. **D.** Example hypnograms for all four monkeys.


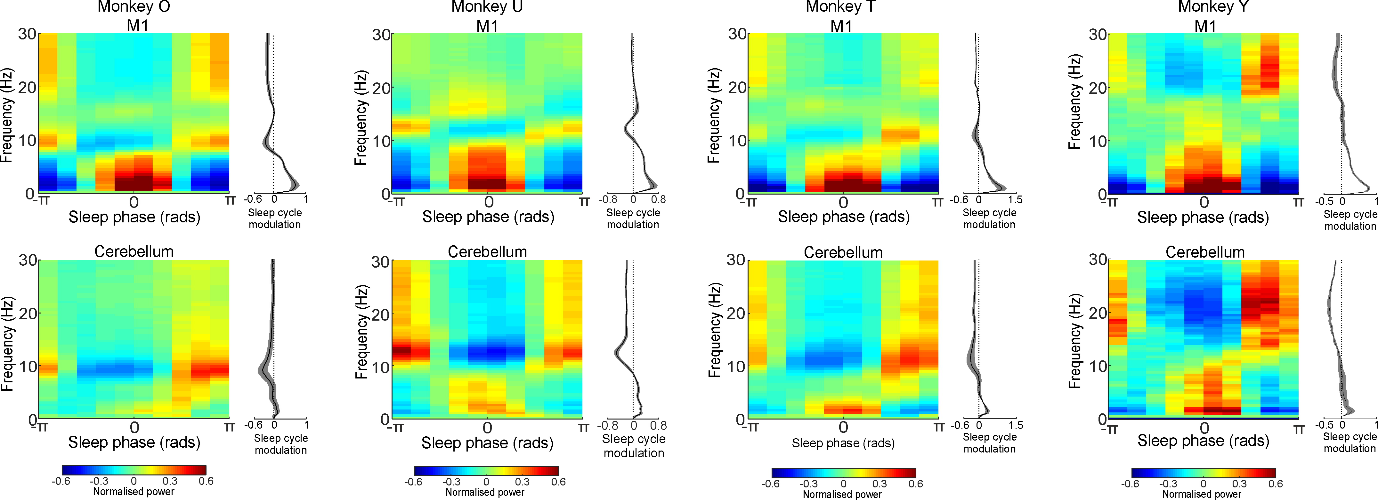


**Supplemental Figure 3. LFP power modulation in M1 and cerebellum during the sleep cycle.** Normalised LFP power as a function of sleep phase, and corresponding sleep cycle modulation. Average of all sessions for 4 monkeys. Shading indicates s.e.m.

**
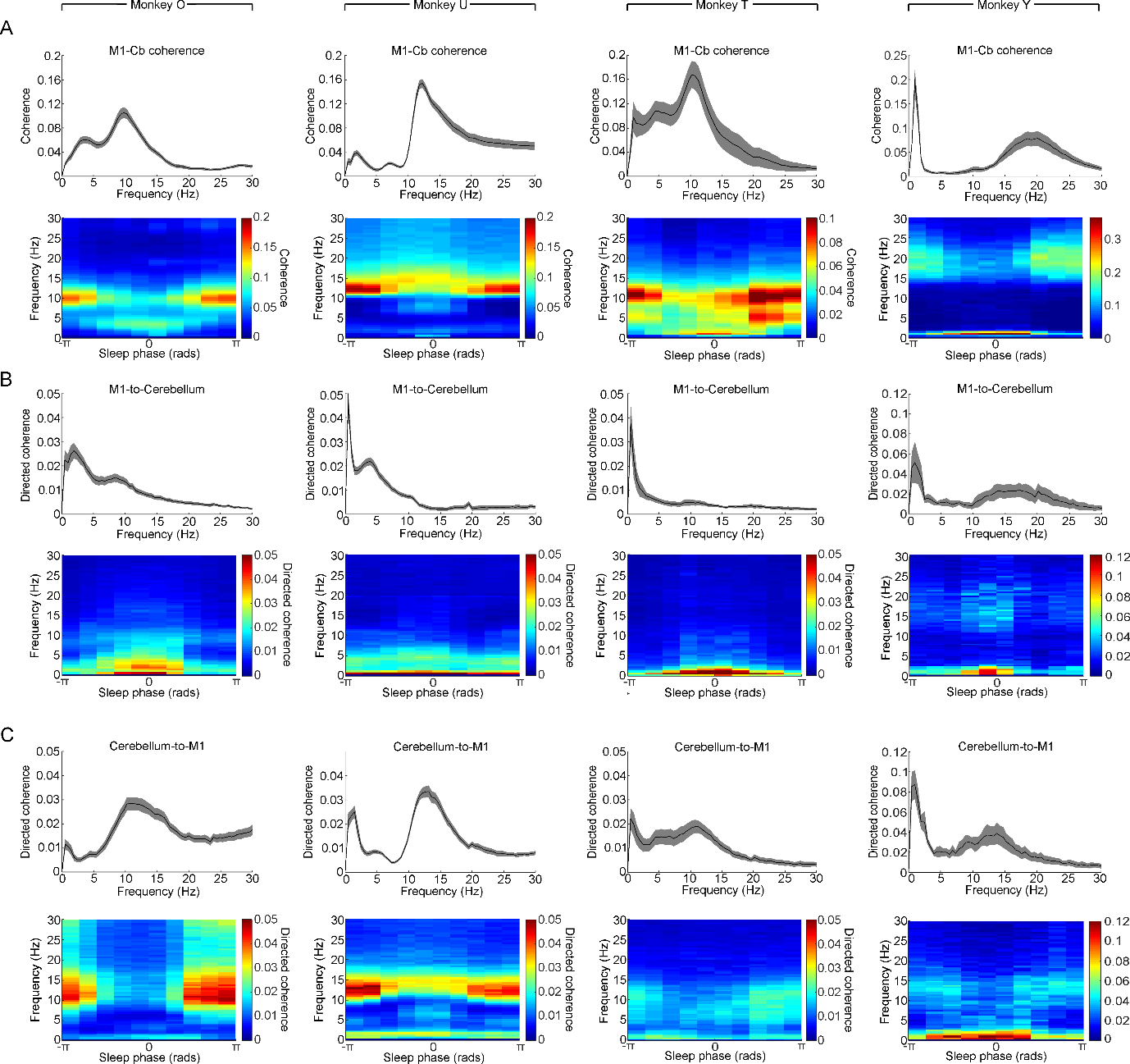
**

**Supplemental Figure 4. Functional connectivity between M1 and cerebellum for all 4 animals. A.** Magnitude-squared coherence between M1 and cerebellum. **B.** Directed coherence from M1 to cerebellum. **C.** Directed coherence from cerebellum to M1. Average of all sessions for 4 monkeys. Shading indicates s.e.m.


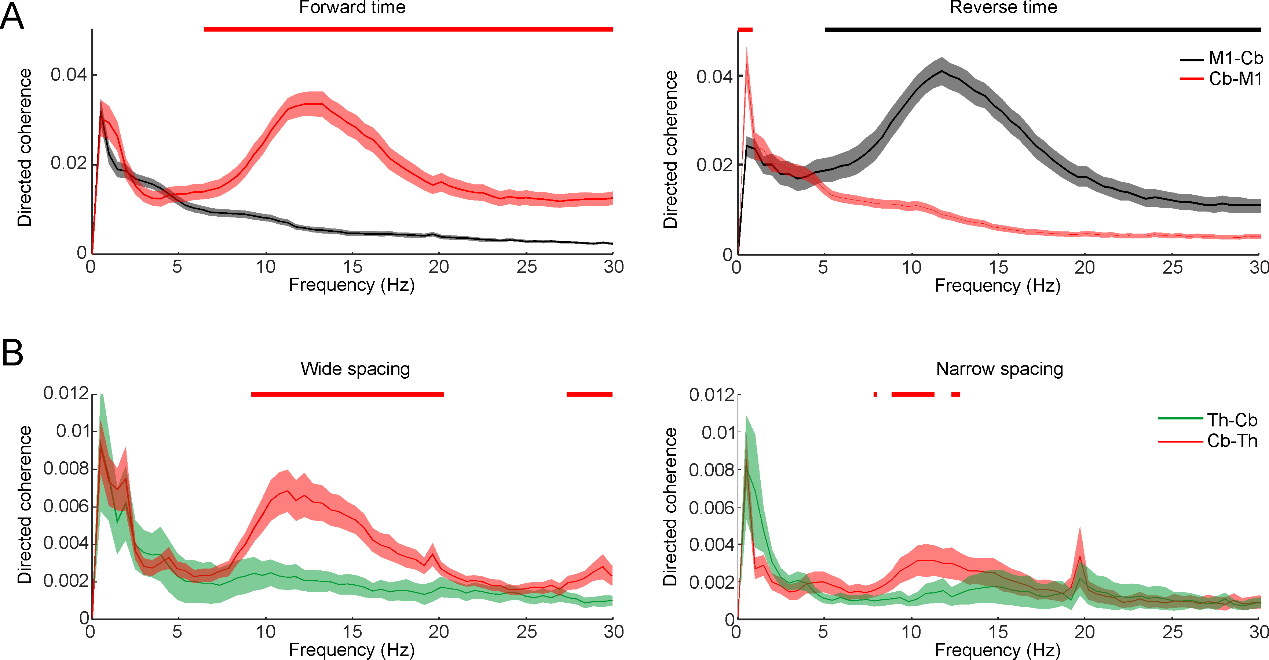


**Supplemental Figure 5. Supplemental directed coherence analyses. A.** Mean directed coherence for forward and time-reversed signals from M1 and cerebellum (averaged across all sessions in all monkeys). Frequencies with significant directionality indicated by coloured lines (P<0.05, paired t-test across sessions). **B.** Directed coherence between cerebellum and thalamus using wide (8.4 mm) and narrow (1.2 mm) spacing between the differential electrode pair for thalamus LFPs (averaged across all session in monkeys T and Y). Frequencies with significant directionality indicated by coloured lines (P<0.05, paired t-test across sessions). Note that the non-significant narrow peak around 20 Hz is a power-line artefact arising from the memory write-cycle in the device, made more prominent due to the smaller signal yielded by differential recording with narrow spacing.


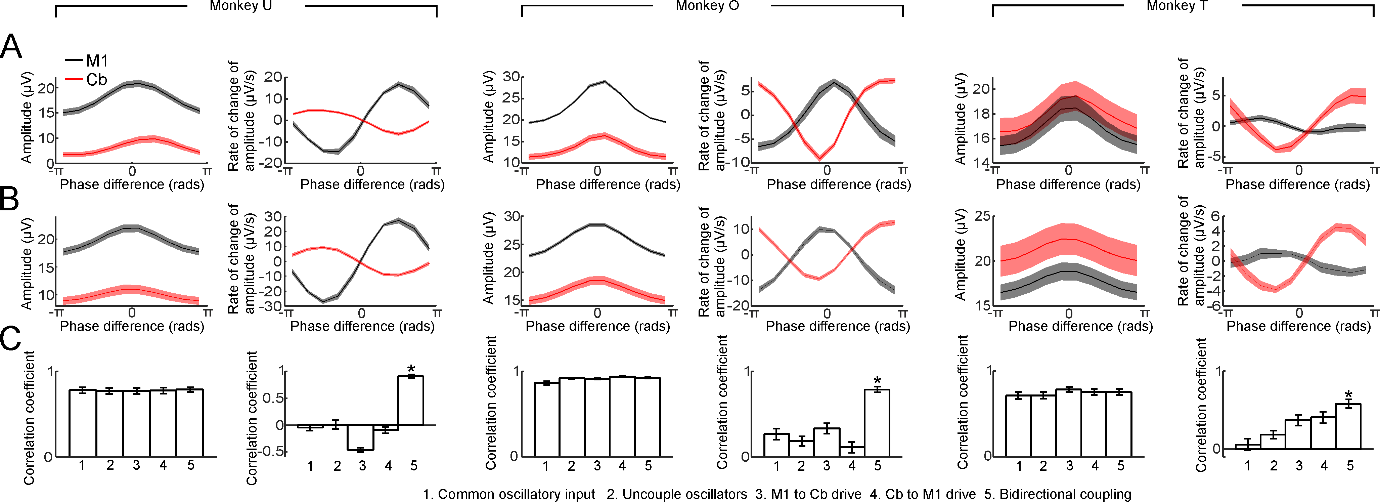


**Supplemental Figure 6. Model fit of spindle amplitude and amplitude derivative vs. phase difference for all animals. A.** M1 and cerebellar spindle amplitude and amplitude derivative against phase difference. **B.** Simulated M1 and cerebellar spindle amplitude and amplitude derivative against phase difference for the full bidirectional coupling model. **C.** Plot of Pearson’s correlation coefficient between experimental and simulated curves for each model. * indicates a significantly higher correlation to all other models (paired t-test). Error bars represent s.e.m. over all sessions.

**Supplemental Table1**

| **Condition** | 1. Common oscillatory input | 2. Uncoupled oscillators | 3. M1-to-cerebellum coupling | 4. Cerebellum-to-M1 coupling | 5. Bidirectional coupling (full model) |
| --- | --- | --- | --- | --- | --- |
| **Monkey O (48 sessions)** |  |  |  |  |  |
| **Mean-squared residual (10^-3^µV^2^, mean±s.e.m.)** | 217 ± 11 | 1.48 ± 0.03 | 1.48 ± 0.03 | 1.48 ± 0.03 | 1.48 ± 0.03 |
| **Pairwise MSR difference from full model**  **(10^-3^µV^2^, mean±s.e.m.)** | 216 ± 11 | 0.0059 ± 0.0007 | 0.0033 ± 0.0004 | 0.0026 ± 0.0003 |  |
| **P value** | 1e-24 | 7e-11 | 2e-10 | 2e-9 |  |
| **Monkey U (39 sessions)** |  |  |  |  |  |
| **Mean-squared residual**  **(10^-3^µV^2^, mean±s.e.m.)** | 243 ± 5 | 2.21 ± 0.04 | 2.20 ± 0.04 | 2.20 ± 0.04 | 2.18 ± 0.04 |
| **Pairwise MSR difference from full model**  **(10^-3^µV^2^, mean±s.e.m.)** | 241 ± 5 | 0.024 ± 0.001 | 0.012 ± 0.00069 | 0.012 ± 0.00065 |  |
| **P value** | 7e-35 | 2e-22 | 2e-19 | 2e-20 |  |
| **Monkey T (29 sessions)** |  |  |  |  |  |
| **Mean-squared residual**  **(10^-3^µV^2^, mean±s.e.m.)** | 97 ± 8 | 0.78 ± 0.04 | 0.78 ± 0.04 | 0.78 ± 0.04 | 0.78 ± 0.04 |
| **Pairwise MSR difference from full model**  **(10^-3^µV^2^, mean±s.e.m.)** | 97 ± 8 | 0.00025 ± 0.00010 | 0.00004 ± 0.00005 | 0.00021 ± 0.00007 |  |
| **P value** | 5e-13 | 0.018 | 0.41 | 0.005 |  |

**Supplemental Table 1.** Mean-squared residual (MSR) under 10-fold cross-validation for the fit of each model to the experimental data. Values are given as mean ± s.e.m. across all sessions. Also shown is the pairwise difference between the residuals for reduced model fits vs. the full model of bidirectional coupling, and the P value of associated paired t-tests across all sessions.
